# Electron ptychographic microscopy for three-dimensional imaging

**DOI:** 10.1038/s41467-017-00150-1

**Published:** 2017-07-31

**Authors:** Si Gao, Peng Wang, Fucai Zhang, Gerardo T. Martinez, Peter D. Nellist, Xiaoqing Pan, Angus I. Kirkland

**Affiliations:** 1 National Laboratory of Solid State Microstructures, College of Engineering and Applied Sciences, Collaborative Innovation Center of Advanced Microstructures and Center for the Microstructures of Quantum Materials, Nanjing University, Nanjing, 210093 People’s Republic of China; 2 Research Center for Environmental Nanotechnology, Nanjing University, Nanjing, 210093 People’s Republic of China; 3 Department of Electrical and Electronic Engineering, Southern University of Science and Technology, Shenzhen, 518055 People’s Republic of China; 4 London Centre for Nanotechnology, London, WC1H 0AH UK; 5 Research Complex at Harwell, Harwell Oxford Campus, Didcot, OX11 0FA UK; 6 Department of Materials, University of Oxford, Parks Road, Oxford, OX1 3PH UK; 7 Department of Chemical Engineering and Materials Science, University of California, Irvine, CA 92697 USA; 8 Department of Physics and Astronomy, University of Califnornia, Irvine, CA 92697 USA; 9 Electron Physical Sciences Imaging Centre, Diamond Lightsource, Diamond House, Oxfordshire, Didcot, OX11 0DE UK

## Abstract

Knowing the three-dimensional structural information of materials at the nanometer scale is essential to understanding complex material properties. Electron tomography retrieves three-dimensional structural information using a tilt series of two-dimensional images. In this paper, we report an alternative combination of electron ptychography with the inverse multislice method. We demonstrate depth sectioning of a nanostructured material into slices with 0.34 nm lateral resolution and with a corresponding depth resolution of about 24–30 nm. This three-dimensional imaging method has potential applications for the three-dimensional structure determination of a range of objects, ranging from inorganic nanostructures to biological macromolecules.

## Abstract (web summary)

Three-dimensional ptychographic imaging with electrons has remained a challenge because, unlike X-rays, electrons are easily scattered by atoms. Here, Gao et al. extend multi-slice methods to electrons in the multiple scattering regime, paving the way to nanometer-scale 3D structure determination with electrons.

## Introduction

Techniques enabling three-dimensional (3D) structure analysis, such as X-ray^1^ or neutron diffraction^2^, and electron microscopy^3^, are invaluable tools for understanding the complex relationship between structure and function in materials. Transmission electron microscopy (TEM) has been extensively used for structural analysis in both physical and biological sciences for many decades, where under carefully defined optical conditions, the structure of 3D objects can be interpreted from two-dimensional (2D) images^3–5^. Electron tomography in the TEM is a similarly well-established method and has been used to solve a large range of complex structures in biology and materials science^6–8^. In the latter discipline, specific examples include Ag clusters^9^, CdTe nanoparticles^10^, morphologically controlled Au rods^11^, and Pt-Co fuel cell material^12^. The success of this method relies on the validity of the projection condition^13, 14^, which allows back projection of 2D images recorded at a series of specimen tilt angles to reconstruct a 3D structure. However, for thicker specimens, the projection approximation can break down primarily as a consequence of multiple interactions of the electrons with the sample. Furthermore, the resolution of a reconstructed tomogram is generally governed by the number of projections and the angular coverage of the data set acquired^15^. For radiation sensitive specimens, the total electron dose budget frequently determines the maximum number of projections that can be recorded, potentially leading to a degradation in the 3D reconstruction resolution, a limitation that is of particular significance for several classes technologically important materials^16, 17^.

Ptychography was originally suggested by Hoppe^18^ and as proposed was based on a 2D multiplicative approximation^19^ to model the interaction of the probe and the specimen. For 3D imaging, ptychography has subsequently been extended to provide projection images using tomographic tilt series in the X-ray regime^13^. Importantly, for X-rays, the projection approximation is generally valid even for a relatively thick specimens at high tilt angles, before significant multiple scattering occurs. In contrast, this approximation breaks down at modest specimen thickness for electrons due to their larger interaction cross section^3^, which leads to multiple scattering. This multiple scattering is often accounted for in image simulations using the multislice method^20, 21^, and an inverse multislice (invMS) method has been recently incorporated into ptychographic reconstruction algorithms, whereby the exit surface wave function of an object is calculated slice by slice as the wave is transmitted through the sample. The feasibility of this approach has been demonstrated for visible light^22, 23^ and X-rays^24^ but not for electrons where it is potentially most valuable due to strong multiple scattering^3^.

Ptychographic coherent diffractive imaging in 2D has been extensively implemented using both visible light^25^ and X-rays^13, 26^. Using electrons, Nellist et al.^27^ first demonstrated atomic resolution in a ptychographic reconstruction using a focused probe. More recently, Pennycook et al.^28^ have reconstructed ptychographic data, also using a focused convergent electron probe by sampling a larger volume of the four-dimensional data set and making use of the entire double overlap regions, where only one scattered disk overlaps with the zero-order disk. Yang et al.^29^ have further advanced this method to larger fields of view by using a high-speed pixelated detector to record the data^30^ and a Wigner distribution deconvolution (WDD)^19^. This work demonstrated that it was possible to obtain 3D structural information from a weakly scattering sample by directly accessing specific depths in the WDD reconstruction. Furthermore, focused probes are compatible with the geometry required for incoherent imaging and so can be used simultaneously to record *Z*-contrast data. An alternative geometry that can be used to retrieve the object transmission function from an array of far-field diffraction patterns is to record these by moving a known, finite-sized (defocused) probe over a sample with partial overlap of the probe positions. This overlap of illuminated regions provides redundancy that allows the iterative phasing algorithm to converge robustly and rapidly^31, 32^. Using this geometry, Humphry et al.^33^ have demonstrated the recovery of the object function of gold nanoparticles at a resolution of 0.236 nm at 30 kV and Putkunz et al.^34^ and D’Alfonso et al.^35^ have extended the attainable resolution to 0.08 nm at 300 kV. Wang et al.^36^ have also demonstrated imaging of B columns in LaB_6_ crystals at a similar resolution with high phase sensitivity using a defocused probe. Importantly, the results reported in these previous studies using both focused and defocused probes make use of a multiplicative approximation. However, this approximation breaks down with increasing specimen thickness, potentially limiting the application of ptychography in studies of many technologically important samples in which significant multiple scattering occurs.

In this paper, we report the use of the invMS approach to retrieve 3D structural information through electron ptychography from graphitized multiwall carbon nanotubes (CNTs) with about 24–30 nm depth resolution and a lateral resolution of 0.34 nm. Furthermore, we use simulations to show that the invMS approach enables depth-resolved sectioning, which is more robust to multiple scattering when compared to the WDD approach.

## Results

### Data acquisition and 3D reconstruction

**Fig. 1:**
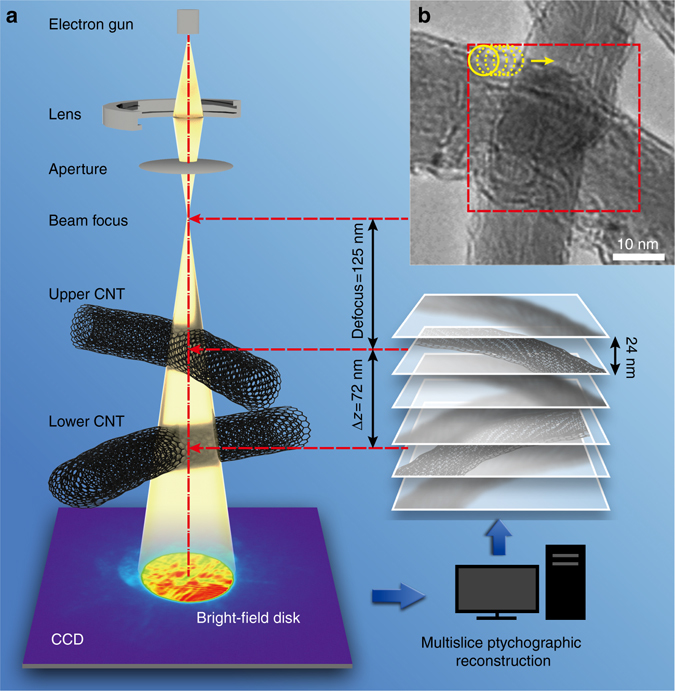
Schematic of the experimental set-up. **a** Optical geometry of the experiment. **b** TEM image of the CNTs; the *red box* indicates the region from which ptychographic data were recorded. The *upper tube* was placed at a distance, df *=* 125 nm below the probe focus. The height separation of the tubes is Δ*z* = 72 nm

The invMS electron ptychography experiment was conducted at 60 kV using a (S)TEM Titan^3^ instrument fitted with a field emission source and probe forming aberration corrector. For the data sets described, the probe convergence semi-angle was 22 mrad. It is important to recognize that in conventional high-angle annular dark field STEM imaging this illumination semi-angle gives a diffraction-limited resolution of 0.11 nm for a focused probe in the absence of effects due to partial spatial coherence^37, 38^. The sample examined consisted of two intercrossing CNTs with a separation of about Δ*z* *=* 72 nm as shown in Fig. 1. This separation was estimated from the condition of minimum contrast^3^ in images of each CNT as shown in Supplementary Fig. 1. The diameters of the upper and lower CNTs were measured as 22 and 18 nm, respectively. The upper tube was placed at a distance, df *=* 125 nm below the probe focus (Fig. 1). A ptychographic data set was subsequently acquired with the incident probe rastered over the region of the sample indicated in Fig. 1b in a grid of 20 × 20 positions with a step size of 1.6 nm. A subarray of 6 × 6 diffraction patterns from the data set is shown as an example in Supplementary Fig. 2. An initial estimate of the illumination wavefield incident on the first slice, which is defined at 24 nm above the uppermost CNT is shown in Supplementary Fig. 3. The method used to estimate the initial probe is described in Supplementary Note 1.

**Fig. 2:**
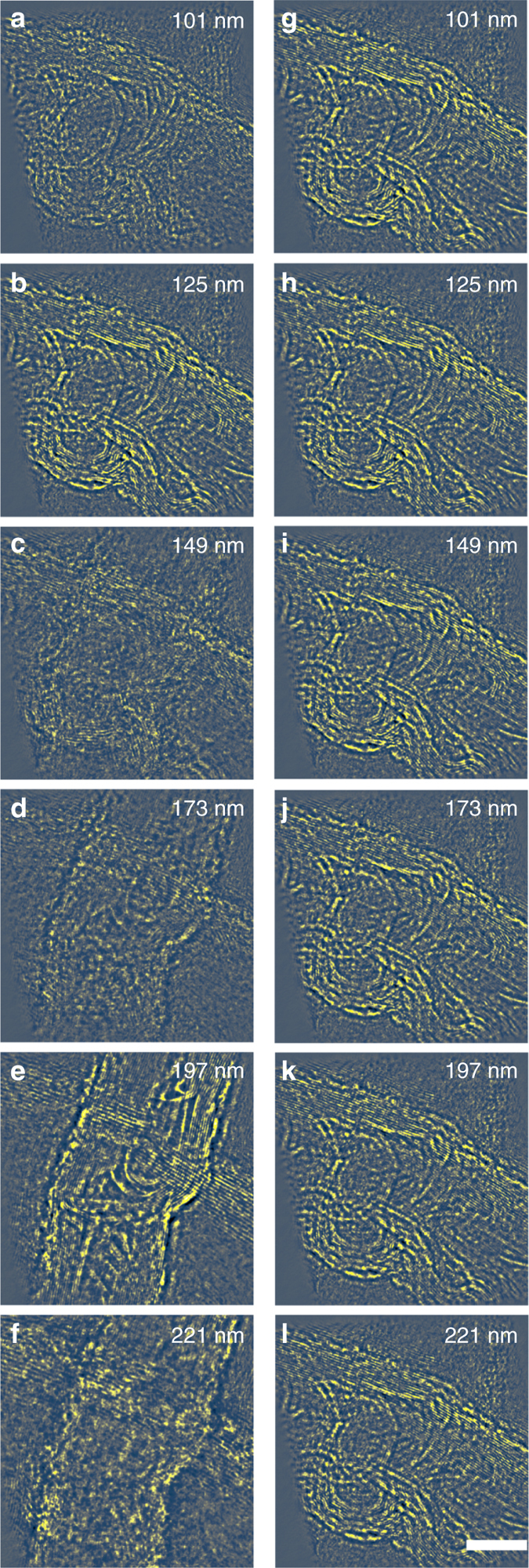
Comparison between reconstruction and propagation effects. **a**–**f** Reconstructed phases at six positions along the optical axis within the sample. **b** and **e** show slices where the upper and lower CNTs are at the Gaussian focus, respectively. Pixel size = 17 pm; image size = 2400 × 2400 pixels. **g**–**l** Fresnel propagation of the reconstructed complex object image phase in **b** to the appropriate sample height. The invMS method shows a depth sensitivity different from that due to simple Fresnel propagation of the reconstructed exit wave function. For example, the lower CNT, becomes visible using invMS optical sectioning in **e**, but remains invisible after applying Fresnel propagation in **k**. The depth value at the *top-right* corner in each figure is defined as the distance from the probe focal point. The 10 nm scale bar is the same for all figures

Figure 2a–f shows the reconstructed phase from each of six slices, separated along the incident beam direction by 24 nm with a pixel size of 17 pm and an image field of view of 2400 × 2400 pixels. The corresponding modulus from six slices of the two CNTs are numbered and displayed sequentially (Supplementary Fig. 4). Figure 2b, e shows the highest contrast, corresponding to the optimal focal planes for the upper and lower CNTs, respectively. An animation of the full stack of reconstructed phase images can be found in Supplementary Movie 1. It should be emphasized that this is a true optical sectioning effect enabled by 3D ptychography using the invMS approach and is not equivalent to a Fresnel propagation of the exit wave as in phase contrast high-resolution TEM^39^. As shown in Fig. 2g–l, Fresnel propagation of the complex transmission function retrieved using a ptychographic reconstruction at depths of 125 nm, where the contrast for the upper CNT is highest, does not recover the phase of the lower tube located at a different depth. Therefore, each recovered phase from 3D ptychogaphical optical sectioning provides a unique identification of the 3D structure at a particular depth.

The 3D ptychographic reconstruction described allows optical sectioning with potential applications to the removal of out-of-focus features in conventional 2D ptychographic imaging as illustrated in Supplementary Fig. 5. The normalized root-mean-square error metric was calculated in the diffraction plane per iteration (Supplementary Fig. 6), which shows rapid convergence. Furthermore, the experimental geometry required is relatively simple with no requirement for mechanical tilting of the sample as in electron tomography^13, 14^ or accurate scanning at different depths along the optical axis as in scanning confocal electron microscopy^40, 41^. However, it may still be useful to combine the approach described with specimen rotation to recover 3D data from very strongly scattering specimens at high resolution, for example, reconstruction of micron thick biological samples^42^, although we note that recent advances in model-based tomographic reconstructions can negate the need for back projection^43^. Although the depth resolution demonstrated in these initial results as described is not yet comparable to that obtained using electron tomography^44^, the data acquisition process is faster and minimal data post processing is required to recover quantitative 3D information. With a larger convergence angle, for example, 71 mrad as previously reported^45^, a depth resolution of 1.9 nm could be achieved. Potentially, the greatest advantage of this method lies in its compatibility with in situ sample holders, which opens up the possibility of quantitative 3D phase reconstruction for samples in liquid^46^, gas^47^, or cryo^48^ environments, without the requirement for a wide gap objective lens pole piece suitable for tomography.

**Fig. 3:**
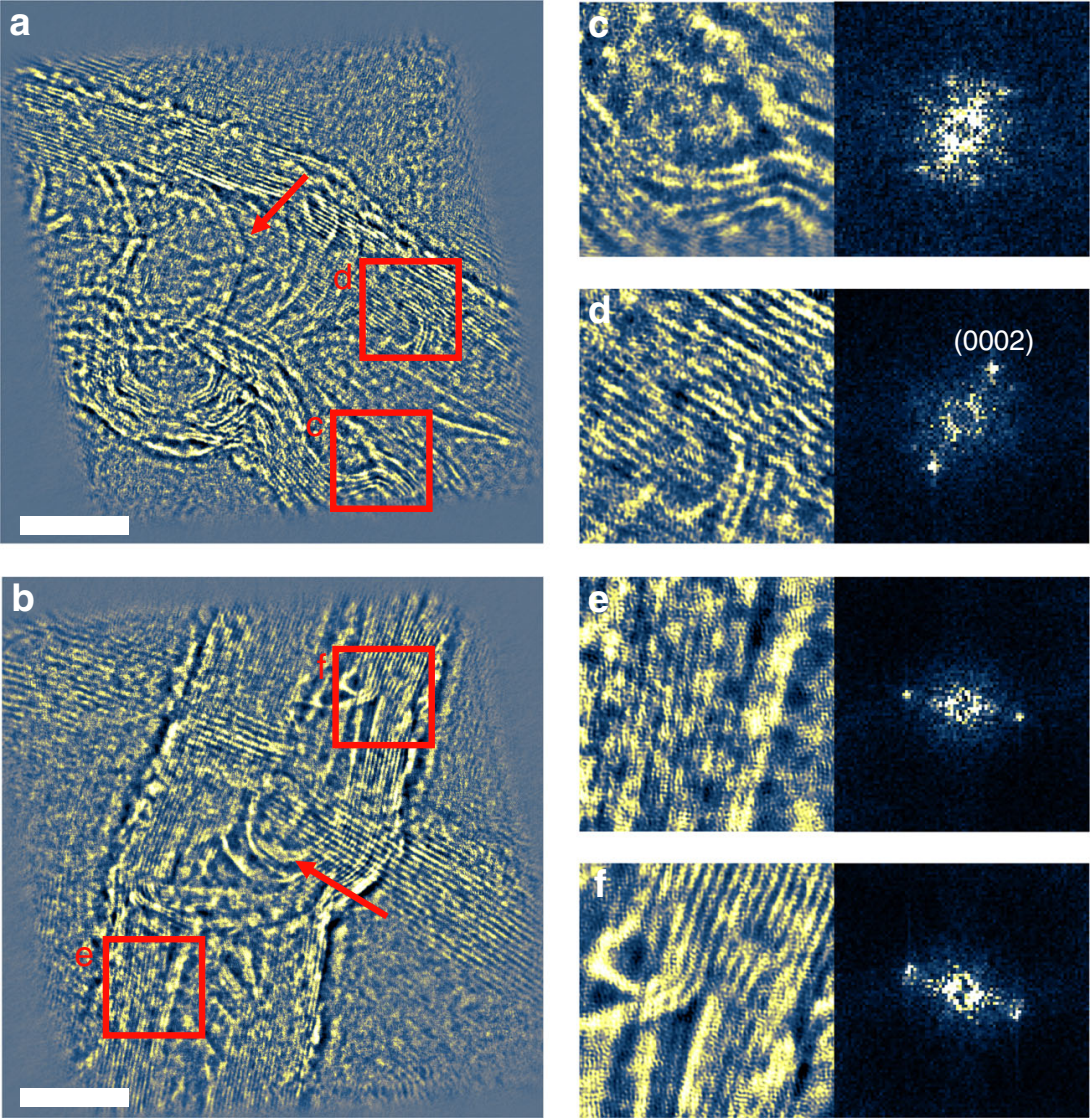
Reconstructed phase in focus. **a**, **b** Phase of the upper and lower CNTs at Gaussian focus, respectively. **c**–**f** Magnified views of regions indicated in **a** and **b** together with calculated power spectra. A lattice spacing of 0.34 nm in both CNTs is evident in both image and spectrum. *Arrows* indicate the compartment layers of the interior tube. Pixel size is 17 pm and the image size of **a** and **b** is 2400 × 2400 pixels. The 10 nm scale bar is the same for **a**–**b**

Ptychographic reconstruction also enables post-reconstruction numerical focusing. Figure 3a, b shows the reconstructed phases of two CNTs at depths of 125 and 197 nm, respectively. The tube walls show lattice fringes at 0.34 nm and compartment layers (indicated by arrows) are observed in the interior of the tubes, which suggests that they are bamboo-shaped multiwalled CNT structures^49^. Figure 3c-f shows magnified views of subregions indicated by the red squares in Fig. 3a, b, respectively, together with their associated power spectra. In Fig. 3c, the power spectrum shows three pairs of reflections arising from the bent tube with a spacing of 0.34 nm. All power spectra shown in Fig. 3c-f calculated from four subregions in both slices show the principal (0002) reflection corresponding to the wall spacing of 0.34 nm^50^. However, as the CNTs are curved and misoriented with respect to the incident beam direction, other higher-order reflections are not resolved^51^.

An additional advantage of post-acquisition focusing lies in the fact that it readily provides data at an optimal defocus, which is beneficial for imaging beam sensitive materials where it is challenging to identify the optimum defocus under low-dose conditions^52^. Hence, this 3D feature of electron ptychography may facilitate low-dose 3D imaging of beam-sensitive specimens, especially in the life sciences and in studies of soft matter. Compared to focused probe experiments^53, 54^, the pixel size and image size of the reconstructed image in the current ptychographic geometry is dependent on the sampling of the diffraction patterns at the detector plane, the illumination size and the number of scanning positions. In our current work as reported, we have reconstructed a series of 2400 × 2400 pixel images with a pixel size of 17 pm from an array of 20 × 20 diffraction patterns. With current and proposed high-speed, high-sensitivity detectors operating at 1000–10,000 frames per second^55^, it should be possible to record the same data at time intervals corresponding to important chemical dynamic processes, for example, in catalysis.

### Theoretical lateral and depth resolutions

To theoretically describe both lateral resolution and depth sectioning, the Ewald sphere construction for the optical configuration was used as shown in Supplementary Fig. 7 (see Supplementary Note 2 for details). The lateral and depth resolution in ptychography^24^ with an incident plane wave along the optic axis are given as

1 $${r_x}{\rm{ = }}{r_y}{\rm{ = }}\frac{1}{{{k_{x,{\rm{max}}}}}}{\rm{ = }}\frac{\lambda }{{{\rm{sin}}\left( {{{\it{\Theta }}_{\max }}} \right)}},{r_z}{\rm{ = }}\frac{{\rm{1}}}{{{k_{z,{\rm{max}}}}}}{\rm{ = }}\frac{\lambda }{{2{\rm{si}}{{\rm{n}}^2}\left( {{{\it{\Theta }}_{\max }}{\rm{/2}}} \right)}}$$

where *r*_*x*_, *r*_*y*_, and *r*_*z*_ represent the spatial resolution in the *x*, *y*, and *z* directions, respectively, *Θ*_max_ is the maximum scattering angle used in the reconstruction and *λ* is the electron wavelength. *k*_*x*,max_ and *k*_*y*,max_ define the maximum lateral distances on the Ewald surface. In the experiments described, a convergent incident beam was used. Due to the low signal-to-noise ratio outside the bright-field (BF) disk at the detector plane, only the central BF disk was used for reconstruction. With curved illumination as in this experiment, the effective maximum scattering angle is twice as large as that of the BF disk in the *k*_*x*_ and *k*_*y*_ directions^27^. Therefore, the boundary of the contrast transfer function for 3D ptychography in reciprocal space using a convergent incident beam with a semi-angle, *α* for scattering in the BF disk can be written (as also illustrated in Supplementary Fig. 8) as:

2 $${k_z}{\rm{ = }}\left\{ {\begin{array}{*{20}{c}}\\ { \pm \left( {\frac{{{\rm{cos}}\alpha - \sqrt {1 - {{\left( {\lambda {k_x} - \sin \alpha } \right)}^2}} }}{\lambda }} \right),\quad {k_x} \ge 0} \\ \\ { \pm \left( {\frac{{{\rm{cos}}\alpha - \sqrt {1 - {{\left( {\lambda {k_x}{\rm{ + }}\sin \alpha } \right)}^2}} }}{\lambda }} \right){\rm{,}}\quad {k_x}{\rm{ < }}0} \\ \end{array}} \right.$$

with lateral and depth resolutions given by

3 $$r_x^\prime {\rm{ = }}r_y^\prime {\rm{ = }}\frac{1}{{{k_{x,{\rm{max}}}}}}{\rm{ = }}\frac{\lambda }{{2{\rm{sin}}\left( \alpha \right)}}$$

and

4 $$r_{z,{k_x}}^\prime {\rm{ = }}\frac{1}{{{k_{z,{k_x}}}}}{\rm{ = }}\frac{\lambda }{{\left| {\cos \alpha - \sqrt {1 - {{\left( {\lambda \left| {{k_x}} \right| - \sin \alpha } \right)}^2}} } \right|}}\quad {\rm{for}}\,{\rm{a}}\,{\rm{given}}\,{k_x}$$

In our experiment, *α* = 22 mrad and *λ* = 4.87 pm at 60 kV yielding lateral resolution $r_x^\prime {\rm{ = }}r_y^\prime {\rm{ = }}0.11$ nm, which are, improved by a factor of two compared to the limit defined by Eq. 1. Apart from the resolved (0002) reflection with a 0.34 nm spacing, our experimental reconstructions did not resolve other higher-order reflections, which might be due either to the fact that the CNTs are curved and misoriented with respect to the incident beam direction^51^ or to the effects of partial coherence and other instrumental instabilities^56^. However, to accurately evaluate the lateral resolution, a sample consisting of 3D CNT bundles is not optimal when compared to single crystals with precisely defined lattice spacings (for example, Si and LaB_6_)^36, 38^. For this reason, further detailed investigations of both experimental lateral and depth resolutions achievable using this method are currently in progress.

**Fig. 4:**
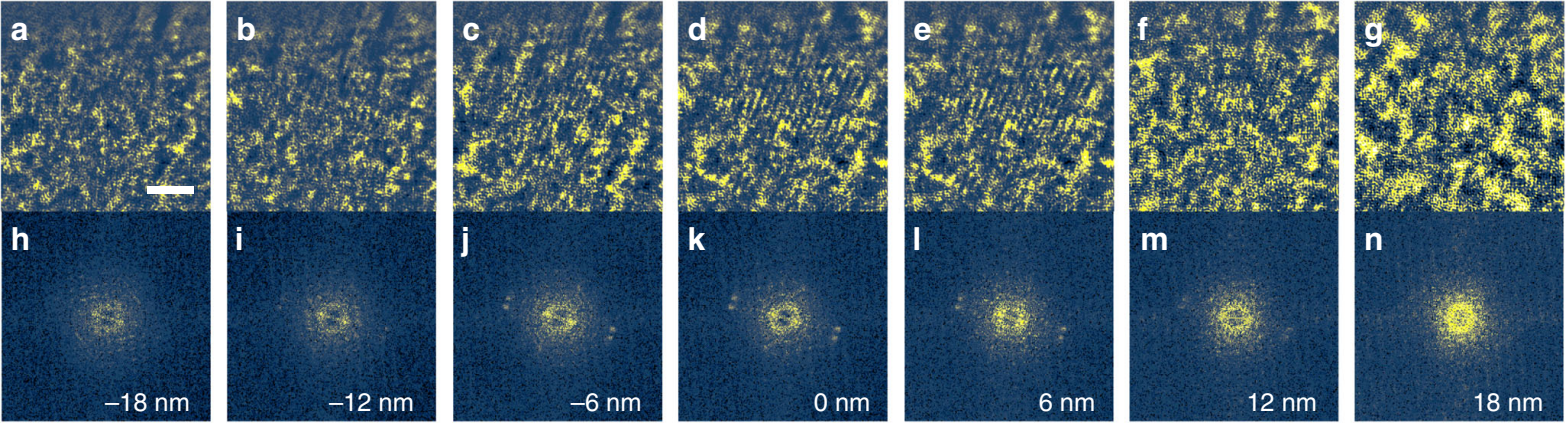
Evaluation of depth resolution. **a**–**g** Reconstructed phase using seven slices from a 21-slice reconstruction with a step of 6 nm along the optical axis and **h**–**n**, corresponding power spectra. Lattice fringes, (0002) with a spacing of 0.34 nm and their corresponding reflections fade outside a sample height range from −12 to 12 nm. This confirms a depth resolution of about 24–30 nm for the spatial frequency corresponding to the 0.34 nm lattice spacing. The 2 nm scale bar is the same for **a**–**g**. The values in **h**–**n** are defined as the distance from the focus plane of the lower CNT, which is at depth of 197 nm

Equation 4 indicates that depth resolution in real space depends on the inverse of the thickness in the region of accessible reciprocal space for a given spatial frequency *k*_*x*_. To evaluate depth resolution experimentally, the region of the CNT bundle showing (0002) lattice fringes with a spacing of 0.34 nm was used in a reconstruction with a finer slice separation of 6 nm as shown in Fig. 4. According to Eq. 2, the boundary of *k*_*z*_ for the spatial frequency corresponding to the (0002) reflection gives a depth resolution of 22.9 nm, which is consistent with the experimental observation of a fading in the contrast of both lattice fringes and their corresponding reflections in the power spectrum within a sample height range of about 24–30 nm.

### Comparison of invMS and WDD approaches

To demonstrate that the primary benefit of invMS ptychography compared to the WDD used by Yang et al.^29^ is the explicit handling of multiple scattering in thick samples, we have carried out simulations using the multislice method^57^. The simulations show that there is a sample thickness limit even for light element materials, such as that used in the present experiment where the invMS can fully accommodate multiple scattering but where the WDD approach cannot. For the sake of computational cost, we modeled a small area of 3 × 3 nm from the overlapping region as shown in Supplementary Fig. 9. In contrast to the work of Yang et al.^29^, the sample used here consisted of multiwalled CNTs with a thickness of about 24 nm that were further annealed at 3680 °C in an inert atmosphere. This process removes both microstructural defects as well as the catalyst particles within CNTs and enhances their graphitic structure^58^. Therefore, to model the simulated area in the overlapping region, two single crystalline carbon nano-rods (CNRs) with a graphitic structure and a similar thickness of 24 nm each, corresponding to the experimental geometry were constructed (see Supplementary Note 3 for the simulation details). The optical sectioning results are shown in Supplementary Fig. 11, upper panel. The WDD reconstruction shows contrast variations along the depth direction and a noticeable contrast reversal in the reconstructed phase data (Supplementary Fig. 12) for two CNRs, which may suggest that WDD has already failed due to dynamical effects at this sample thickness. For imaging at 60 kV, the example of two 24 nm thick graphite rods used would be expected to exhibit a dynamical scattering behavior as demonstrated by the simulation in Supplementary Fig. 13 and therefore violate the multiplicative approximation. Hence, the contrast reversal observed in WDD optical sectioning is likely due to dynamical scattering. However, this is not the case for reconstructions using the invMS approach. It can be seen that invMS reconstruction recovers more faithful phase data (Supplementary Fig. 11) across various depths and was able to discriminate the two CNRs clearly at different heights. Therefore, while for 2D imaging the WDD has produced impressive results at atomic resolution, its 3D optical sectioning capability for thick samples is limited by multiple scattering. This is consistent with the theory underlying the WDD, which makes use of the multiplicative approximation. For the experimental geometry used here, two < 0001> oriented graphite rods with a 24 nm thickness, the multiplicative assumption appears to break down at 60 kV, although we note that recent results using the WDD provide faithful reconstructions from crystalline samples at 200 kV^59^. Overall, the invMS approach provides depth-resolved sectioning, which is robust to multiple scattering, although the attainable resolution has yet to be shown to be competitive with that achieved using the WDD and a focused probe.

## Discussion

Compared to experiments using a focused probe and an integrating high-speed pixelated detector^30^ as used to record data for the recent WDD work^19, 29^, the defocused probe used in invMS is incompatible with the optical conditions required for annular dark-field imaging. However, despite this disadvantage, the data acquisition described here does not necessarily require a high-speed pixelated detector^30^ as used successfully in the WDD experiments^29^, the speed of which can practically limit the number of probe positions and hence the size of the final reconstruction in pixels. In contrast, the size of the current reconstruction can be as large as 2400 × 2400 pixels at a sampling of 17 pm per pixel.

In future, we anticipate extending this approach by using direct counting electron detectors that are designed to have a very wide dynamic range^60^. Using this type of detector, we expect that much weaker signals can be detected from weakly scattering objects under low-dose conditions and also that the signal in the dark-field region outside the BF disk will be recorded with useable signal to noise ratio. In this geometry, the scattering angle used in the reconstruction will be extended to the maximum collection angle acquired by the detector, which will improve both lateral and depth resolution.

In conclusion, we have demonstrated for the first time that defocused probe electron ptychography using an invMS method can provide depth-sectioned information from a 3D complex transmission function of a thick sample (including both amplitude and phase). The method provides high contrast, quantitative phase maps at close to atomic lateral resolution and with a few tens of nanometers depth resolution. This 3D electron ptychographic method is potentially applicable to in situ TEM experiments as it does not require mechanical tilting of the specimen through large angles as is the case for electron tomography. The additional capability to carry out post-acquisition focusing of the reconstruction coupled with high-dose efficiency^61^ particularly when coupled to high-speed and high-sensitivity detectors^55^ should provide a route to structural determination, using the recovered, fully quantitative ptychographic phase from thicker, radiation sensitive specimens. In the future, we anticipate that this method will find a wide range of applications in 3D structure determination of thick objects, ranging from inorganic nanostructures, heterostructures or ferroic domain structures to biological macromolecules.

## Methods

### Materials

The studies reported used a commercially available graphitized multiwall CNT powder (698849, Sigma-Aldrich), which was produced by chemical vapor deposition and further annealed in an inert atmosphere up to a temperature of 3687 °C. This high-temperature process efficiently graphitized the multiwall CNTs^58^. The powder was first suspended in water and then ultrasonicated for 10 min before being drop-cast directly onto holey carbon-coated TEM grids. In order to disperse the tubes with different geometries, dropping and drying was repeated several times. The two tubes selected for these experiments had a height separation, Δ*z* = 72 nm measured by the minimum contrast for the two tubes as shown in Supplementary Fig. 1.

### 3D ptychography using the invMS approach

The iterative method for reconstruction used the ptychography algorithm with the invMS approach^22^ is summarized below.

The multislice method is widely used in electron microscope simulations. The object is considered as a series of *N* slices, *O*_*n*_(**r**) (*n* *=* 1… *N*), separated by distances Δ*z*_*m*_ (*m* *=* 1… *N* − 1), and the wavefront exiting the specimen is calculated using a series of multiplications and Fresnel propagations between the slices. We label the probe as *P*_1_(**r**−**r**_*j*_), where **r**_*j*_ indicates the relative shift of the probe and object and the subscript 1 indicates that the probe is located at the entrance plane of the first slice of object. The corresponding diffraction pattern acquired in the experiment is labeled, *I*_*j*_.

For the iteration, we use *I*_*j*_ to update both the probe and object, first calculating the exit wave for the first slice of the object as *ψ*_1_ = *P*_1_(**r**−**r**_*j*_) × *O*_1_(**r**). For brevity, we subsequently omit the coordinate, **r**.

The exit wave is subsequently propagated to the entrance plane of the second slice using ${P_2}{\rm{ = Pro}}{{\rm{p}}_{{\rm{\Delta }}{z_1}}}[{{{\psi}}_1}]$, where Δ*z*_1_ is the distance between the first and second slices. These two steps are recursively continued to finally yield the wave at the exit plane of the final slice, as *ψ*_*N*_.

Propagation between slices uses a Fresnel method:

5 $${\rm{Pro}}{{\rm{p}}_{{\rm{\Delta }}{z_1}}}\left[ {{{\rm{\psi }}_1}} \right]{\rm{ = }}{{\cal F}^{ - 1}}[{\cal F}[{{\rm{\psi }}_1}]{\rm{ \times exp}}(i\pi \lambda (k_x^2{\rm{ + }}k_y^2){\rm{ \times \Delta }}{z_1})]$$

where ${\cal F}$ and ${{\cal F}^{ - 1}}$ represent a Fourier transform and inverse Fourier transform, *λ* is wave length, *k*_*x*_ and *k*_*y*_ are coordinates in diffraction plane.

The propagation of *ψ*_*N*_ to the detector plane is carried out through a Fourier transform, ${\rm{\Psi }} = {\cal F}[{{\rm{\psi }}_N}]$. The amplitude is then replaced with the experimental data to yield ${\Psi ^\prime }{\rm{ = }}\frac{\Psi }{{\left| \Psi \right|}} \times \sqrt {{I_j}}$, where the superscript indicates that the value is updated. Inverse Fourier transform gives ${\rm{\psi }}_N^\prime {\rm{ = }}{{\cal F}^{ - 1}}[{{\rm{\Psi }}^\prime }]$.

Using both the updated exit wave ${\rm{\psi }}_N^\prime$ and *ψ*_*N*_, the last slice and the probe at the last slice is updated as:

6 $$O_n^\prime {\rm{ = }}{O_n}{\rm{ + }}\alpha {\rm{ \times }}\frac{{P_n^{\rm{*}}}}{{\left| {{P_n}} \right|_{{\rm{max}}}^2}}{\rm{ \times }}\left( {{\rm{\psi }}_n^\prime - {{\rm{\psi }}_n}} \right)$$

7 $$P_n^\prime {\rm{ = }}{P_n}{\rm{ + }}\alpha {\rm{ \times }}\frac{{O_n^{\rm{*}}}}{{\left| {{O_n}} \right|_{{\rm{max}}}^2}}{\rm{ \times }}\left( {{\rm{\psi }}_n^\prime - {{\rm{\psi }}_n}} \right)$$

where the subscript *n* indicates the slice number, for the last slice, *n* = *N*.

In the second step, we inversely propagate the probe at the last slice to the exit plane of the former slice to get ${\rm{\psi }}_{N - 1}^\prime {\rm{ = Pro}}{{\rm{p}}_{ - {\rm{\Delta }}{z_{N - 1}}}}[P_N^\prime ]$. Following these two steps, all the object slices and probe functions are updated to the first slice, $P_1^\prime ({\bf{r}} - {{\bf{r}}_j})$.

For the next iteration, the probe is moved to the next position in the data set and the newly updated object slice is used as initial estimate.

For the experimental set-up from which data was collected, the slice thickness can be chosen such that the minimum separation makes two neighboring slices of the object lie outside of the bounds of the multiplicative approximation^22^. For a finer slice thickness and a larger total slice number, a greater number of unknown pixels in the specimen reconstruction need to be reconstructed. Therefore, both of these values are also dependent on the degree of the redundancy of the ptychographic data, similar to the over-sampling ratio as described elsewhere^62^.

### Experimental configuration

Data were recorded at 60 kV using an FEI Titan^3^ cubed 60–300 electron microscope fitted with a Schottky field emission source. Figure 1a shows a diagram of the optical configuration used. The probe-forming convergence semi-angle was 22 mrad. The upper CNT was placed at a distance, df *=* 125 nm below the probe focal point and was separated from the lower one by a distance of 72 nm along the optical axis as shown in Fig. 1a. The distance, Δ*z*_1_ between the sample and the probe crossover was subsequently refined using knowledge of the inter-atomic spacings between the walls of the CNTs. This experimental configuration ensures that the extent of the illumination was well defined and gives a probe diameter of about 5.5 and 8.7 nm at the middle planes of the two CNTs, respectively. A scan coil was used to position the beam in a 20 × 20 rectangular grid with a nominal pitch of 1.6 nm as shown in Fig. 1b. In this configuration, the overlap between adjacent positions was calculated to be 72.4 and 82.4% for the slices in which the two CNTs were located, sufficient to fulfill the ptychographic sampling requirement for the probe extent, scan steps, and the detector pixel size used^31^. A 6 × 6 subarray of typical diffraction patterns taken from the 20 × 20 array is shown as an example in Supplementary Fig. 2. From the over-sampling ratio^62^, the degree of the redundancy of ptychographic data used here is estimated as:

8 $$\sigma {\rm{ = }}\frac{{{\rm{Total}}\,{\rm{number}}\,{\rm{of}}\,{\rm{dps}}}}{{2\left( {{\rm{Pixels}}\,{\rm{in}}\,{\rm{specimen}}\,{\rm{and}}\,{\rm{probe}}\,{\rm{reconstructions}}} \right)}}{\rm{ = 5}}{\rm{.9}} \cdot $$

### Data availability

All relevant data are available from the authors on reasonable request.

## Electronic supplementary material

Supplementary Information

Supplementary Movie 1
