# Supplementary material for: Electron ptychographic microscopy for three-dimensional imaging
Source: Nat Commun. 2017 Jul 31;8:163. doi: 10.1038/s41467-017-00150-1 (PMC5537274; doi:10.1038/s41467-017-00150-1)
Supplement: Supplementary file 1 — Supplementary Information [file 41467_2017_150_MOESM1_ESM.pdf]

File Name: Supplementary Information
Description: Supplementary Figures, Supplementary Notes and Supplementary References.

File Name: Supplementary Movie 1
Description: **Reconstructed phases at 6 positions along the optical axis within the sample.** The slice number is labeled at the bottem right corner and the depth value at the top-right corner in each figure is defined as the distance away from the probe focal point.

Supplementary Figures

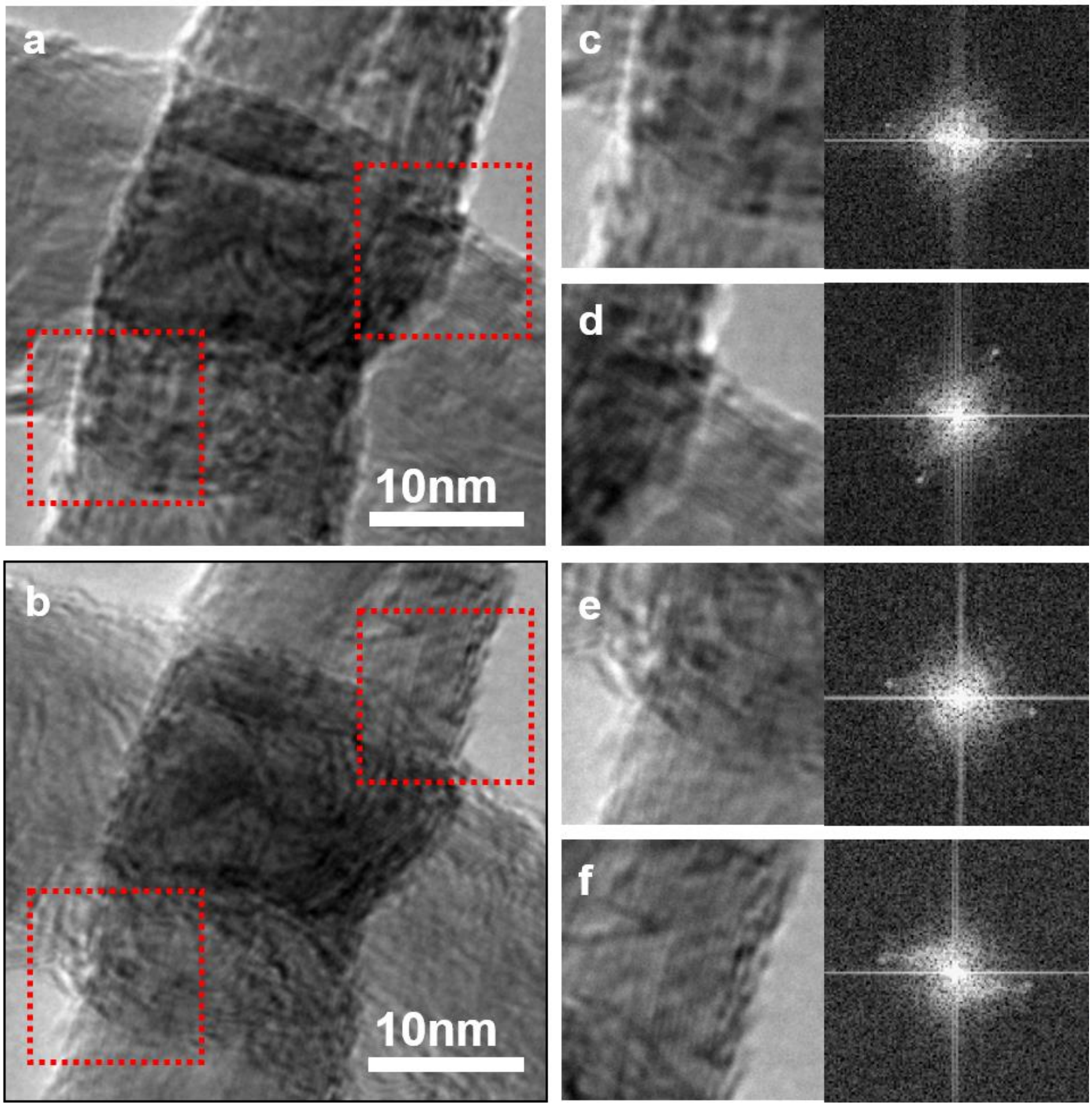

Supplementary Figure 1 | TEM images of the CNTs used for ptychographic reconstruction. **a** and **b**, TEM images taken at the defocus giving rise to minimum contrast for the upper and lower CNTs, respectively. **c**-**f**, Magnified images and their calculated power spectra from areas indicated in **a** and **b**.

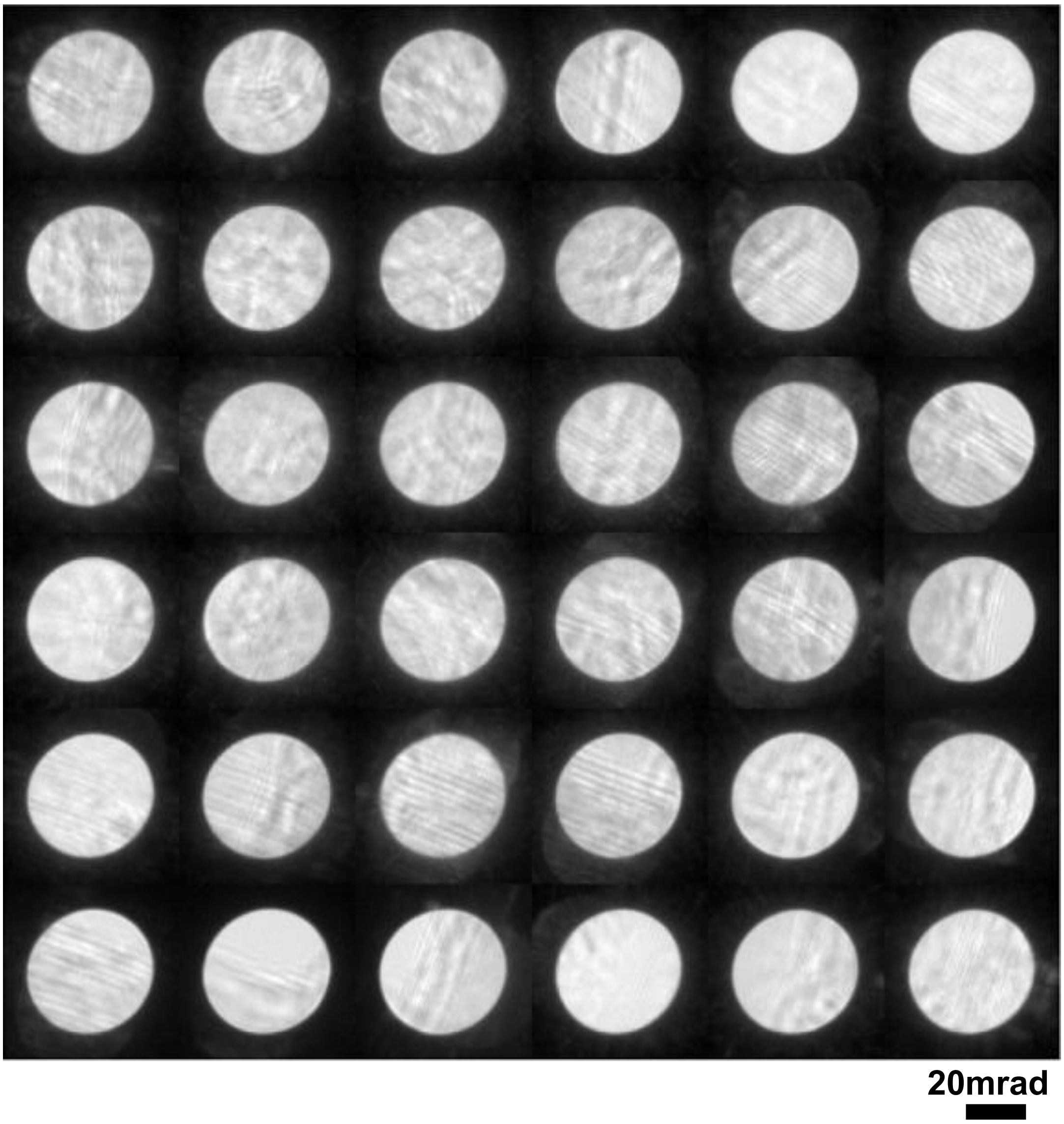

Supplementary Figure 2 | Typical diffraction patterns. Diffraction patterns in a 6 × 6 sub-array from a total array of 20 × 20 patterns recorded with the probe focal point at 125 nm above the upper CNT.

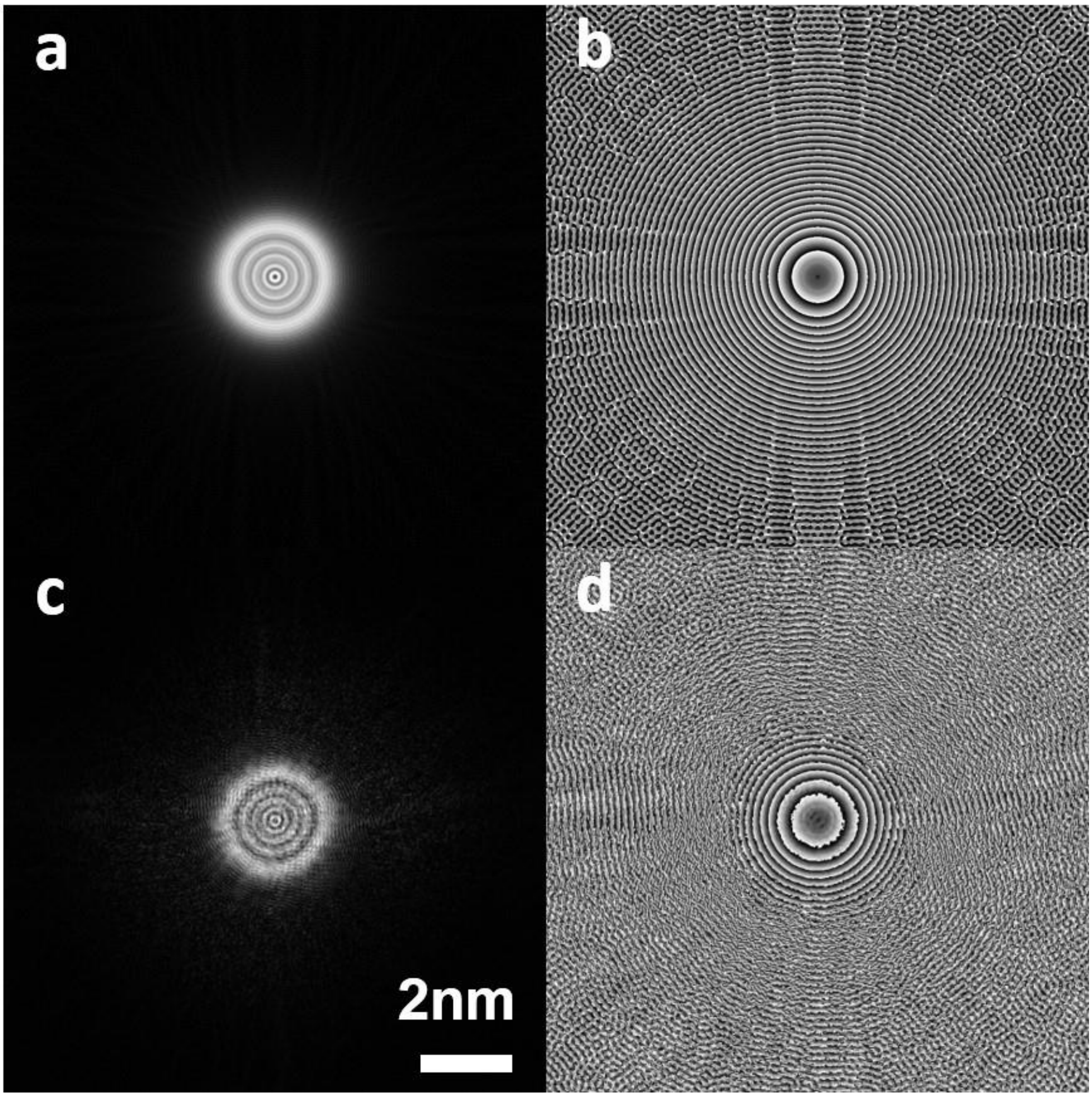

Supplementary Figure 3 | Estimated and reconstructed probe functions. a, Modulus and **b**, Phase of the initial estimated probe function for the first slice at df = 101 nm. **c**, Modulus and **d**, Phase of the probe function after reconstruction.

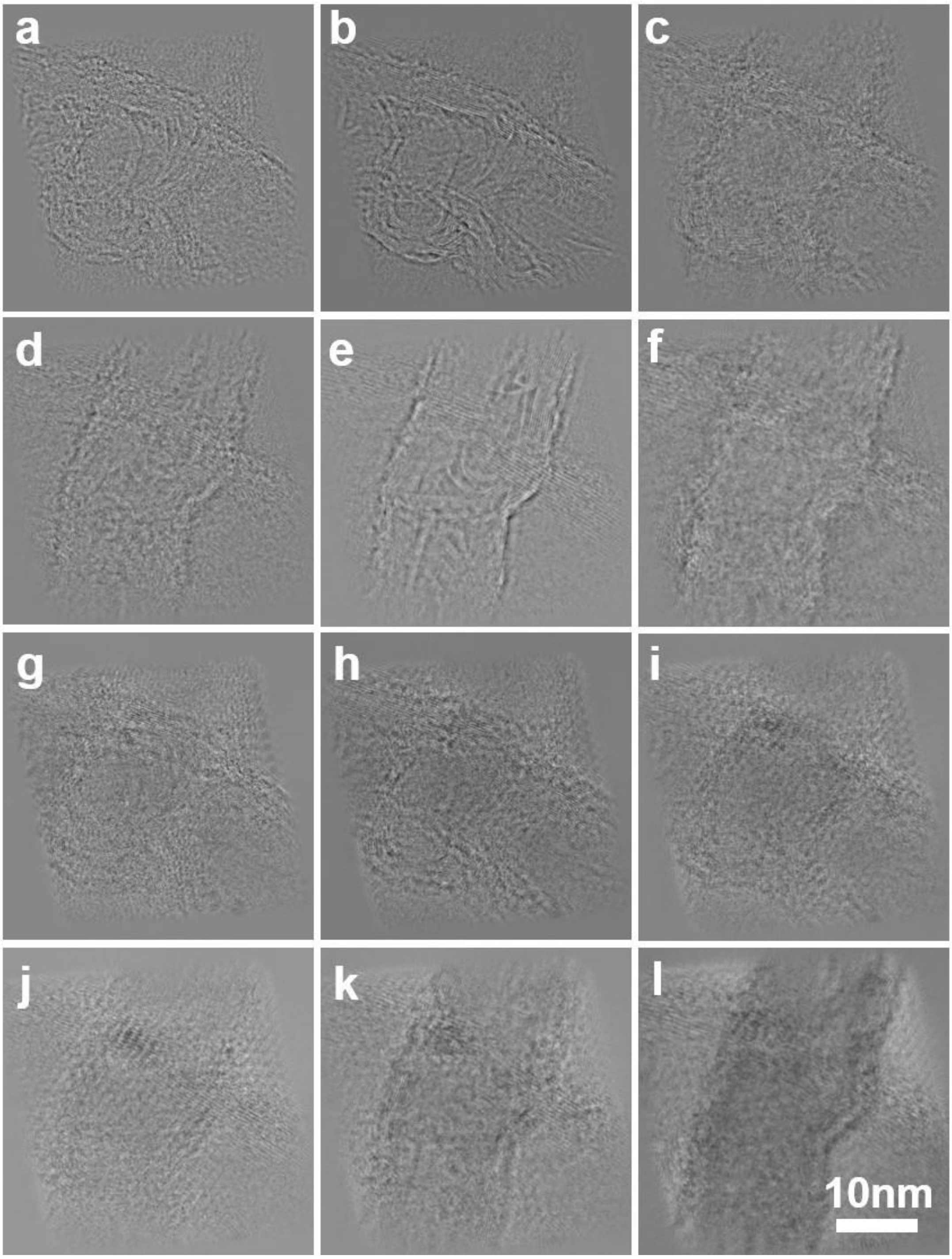

Supplementary Figure 4 | Six slices of the ptychographic reconstruction. a-f, Phase and **g-l** Modulus of two CNTs with a step size along the incident beam direction of 24 nm. The scale bar is the same for all figures.

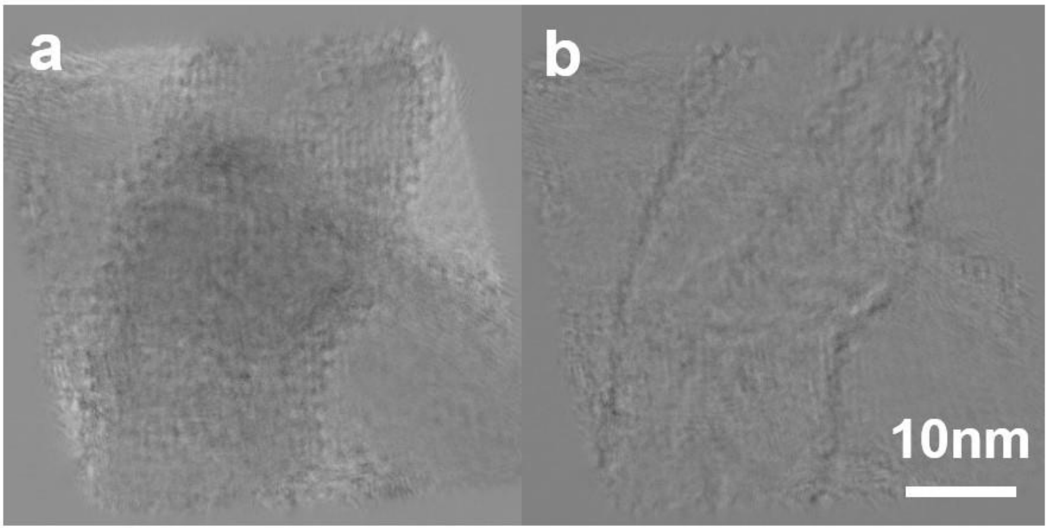

Supplementary Figure 5 | A 2D ptychographic reconstruction. **a**, Phase and **b**, Modulus of two CNTs.

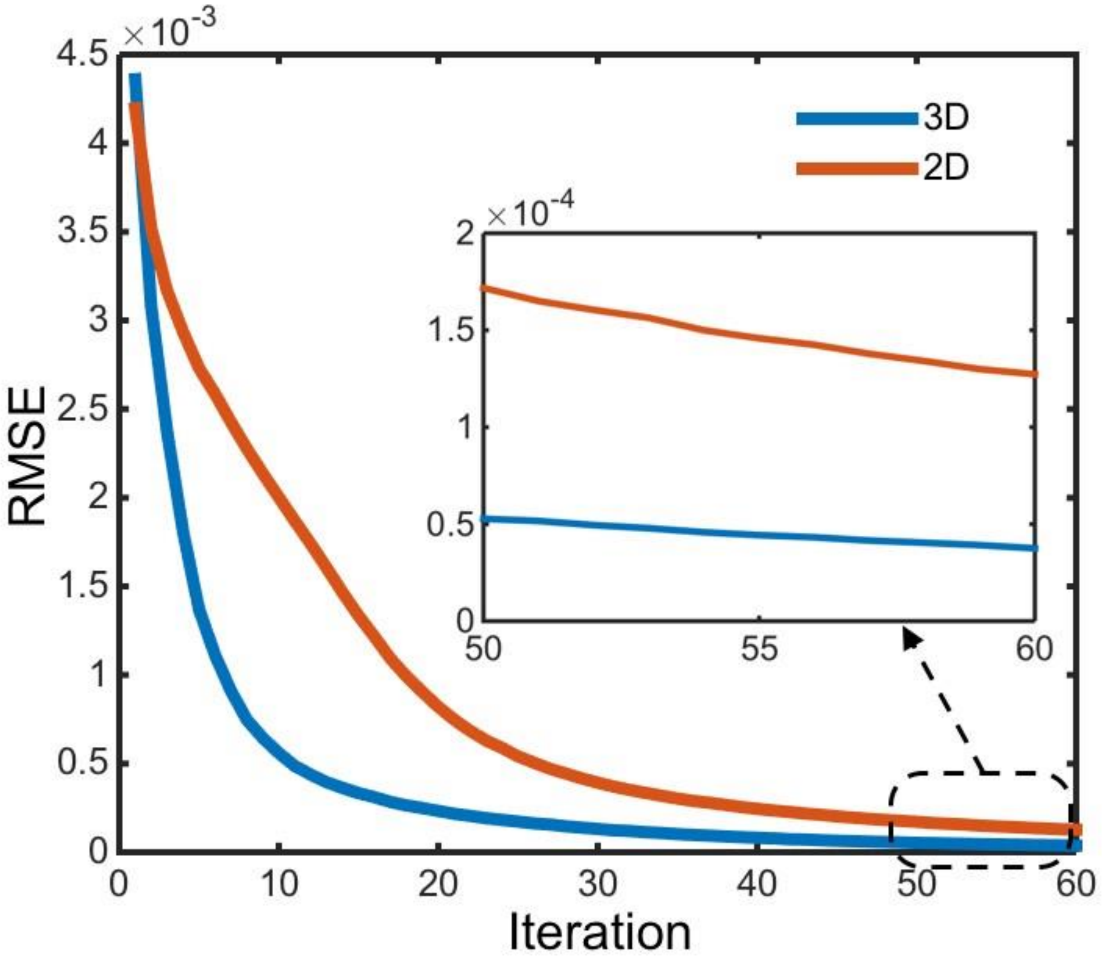

Supplementary Figure 6 | Root Mean Square Error (RMSE). RMSE for 3D (—) and 2D (—) reconstructions, respectively.

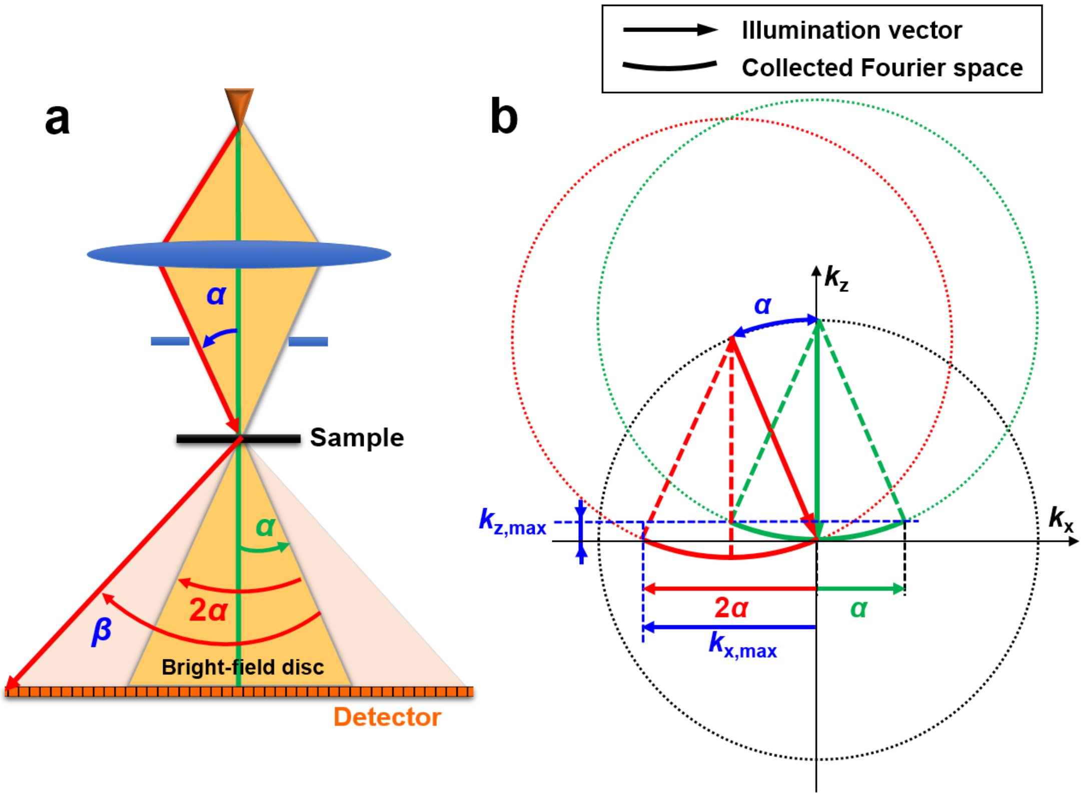

Supplementary Figure 7 | Schematic illustration of extended resolution using convergent illumination. a, Schematic of the experimental geometry for convergent illumination with a convergence semi-angle, α. **b**, Ewald sphere construction for rays incident along the optical axis (—) and at an angle α (—). Since only the central BF disks of the diffraction patterns were processed in this work the maximum effective scattering angle was twice that of the incident semi-angle.

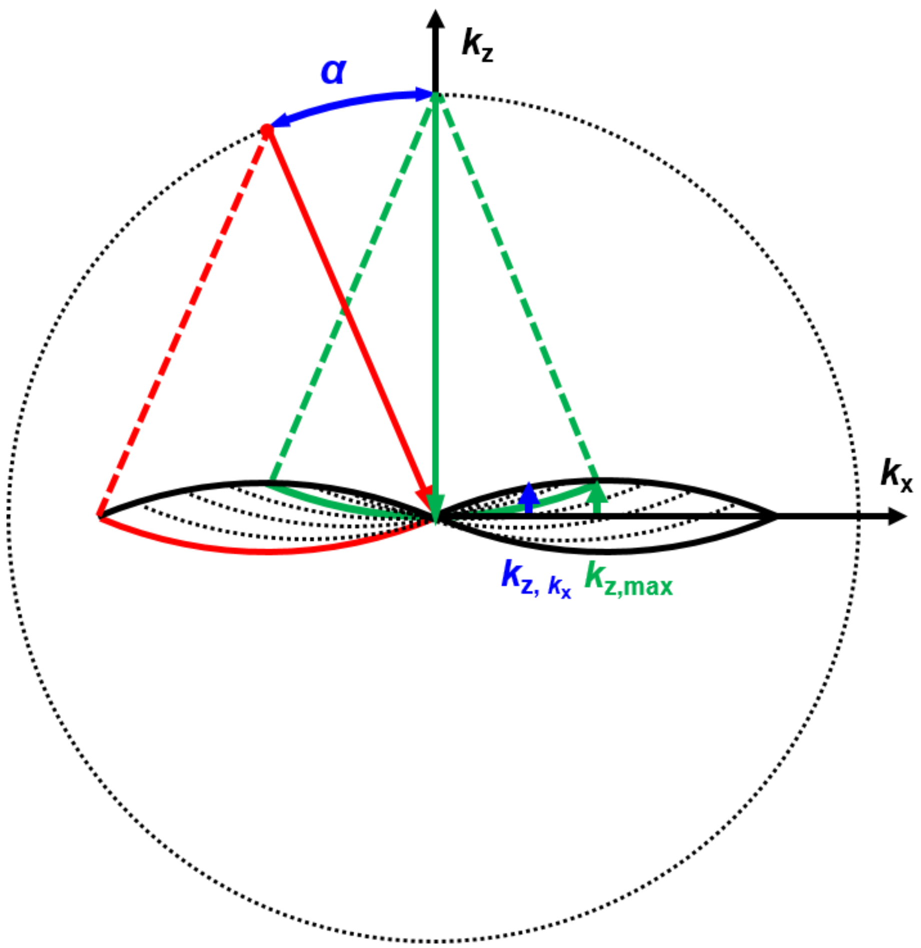

Supplementary Figure 8 | Schematic illustration of the boundary of the coherence transfer function (CTF) in reciprocal space. For the **k** vector (green arrow) of an incident plane wave along the optic axis allowed scattering vectors must lie on the Ewald sphere (green solid arc). For a tilted incident **k** vector, the Ewald sphere is defined by the dotted line. The cone of scattered wave vectors admitted by the bright field detector remains constant, selecting a series of spherical caps in 3D reciprocal space. The Ewald sphere for the **k** vector with a maximum tilt angle α (red arrow) gives a boundary (red arc) to the CTF, which can be expressed by Supplementary Equation 3 as a function of k_x.

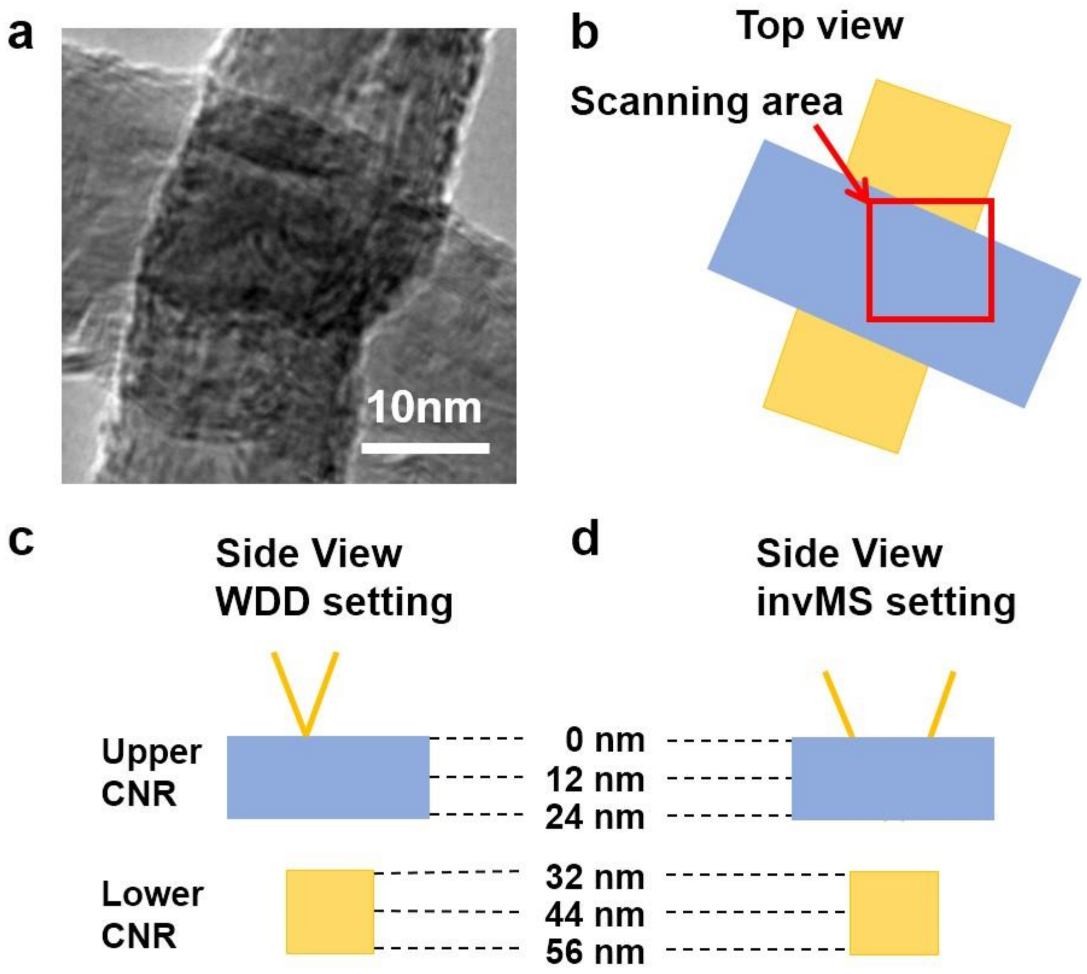

Supplementary Figure 9 | Simulation configurations for WDD and invMS ptychography. **a**, Experimental TEM images of the two crossed CNTs. **b**, Schematic configuration (Top view) of the two crossed CNRs. The red box indicates the reconstruction area in the simulations. The crystal model of the CNRs has graphitic AB stacking structure. **c** and **d**, Schematics (Side view) of the simulation geometries with a focused probe for WDD and defocussed probe for invMS ptychography, respectively.

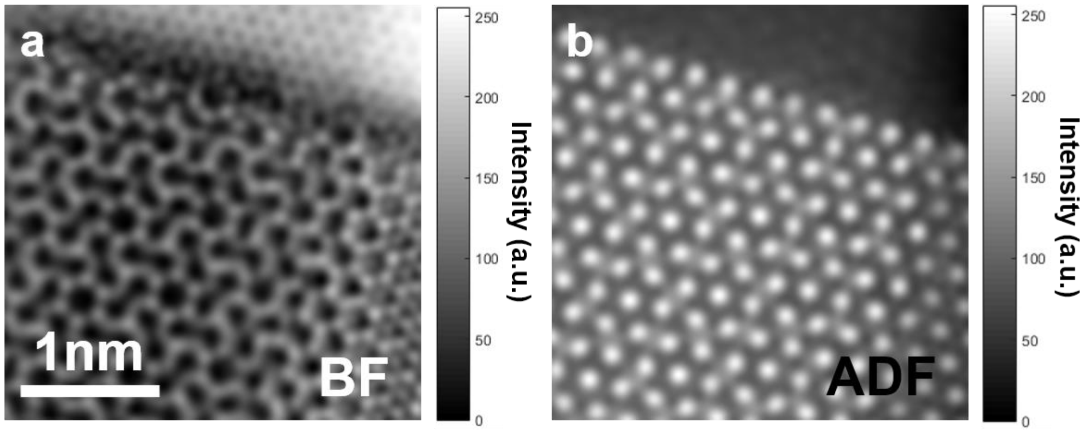

Supplementary Figure 10 | Synthetic BF and ADF images. a and **b**, Synthetic BF and ADF images calculated from the diffraction patterns simulated for the geometry for WDD as shown in Supplementary Fig. 9c.

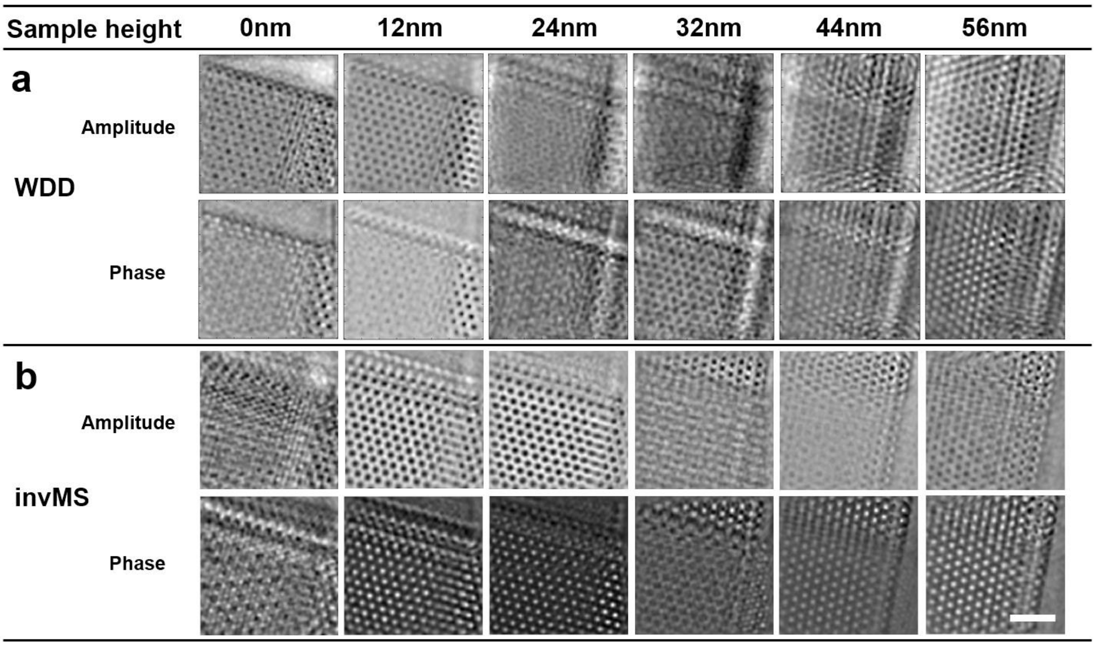

Supplementary Figure 11 | Amplitude and phase at different sample heights reconstructed using WDD and invMS ptychography. Six slices of phase and amplitude reconstructed at the top, middle and bottom planes of the two CNRs, using WDD (**a**) and invMS ptychography (**b**), respectively. These slices are at the distance of 0, 12, 24, 32, 44 and 56 nm from the top surface of the upper CNR as shown in Supplementary Fig. 9 c and d. The scale bar is 1 nm for the figures.

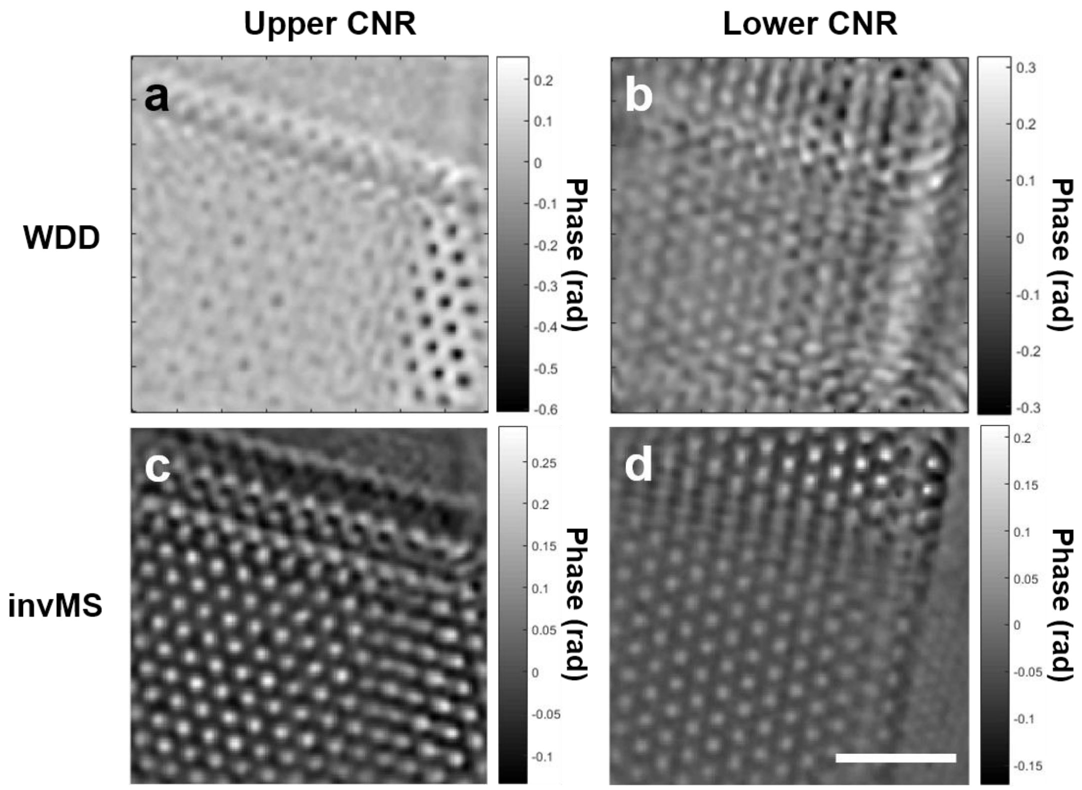

Supplementary Figure 12 | Comparison of the enlarged phases recovered. a and **b**, Phases reconstructed at the middle plane of the upper and lower CNRs using the WDD ptychography, respectively. **c** and **d**, Phases reconstructed at the middle plane of the upper and lower CNRs using the invMS. The scale bar is 1 nm for all figures.

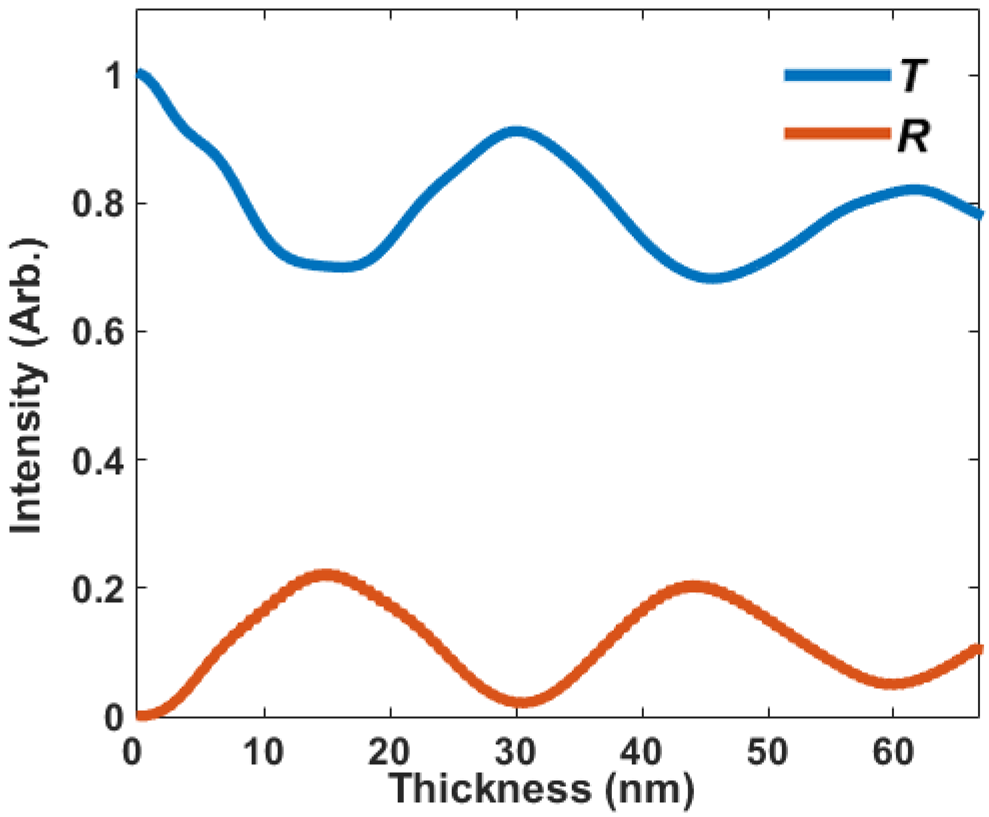

Supplementary Figure 13 | Dynamical scattering behavior in a <0001> orientated single crystalline graphite sample with AB stacking. Thickness dependence of the transmitted (*T*) and Bragg-reflected intensity (*R*) are calculated using non overlapping CBED disks for a <0001> orientated single crystalline graphite sample. The Bragg reflected intensity is a sum of the intensity of the six 1st order reflections; $(11\bar{2}0), (\bar{1}\bar{1}20), (1\bar{2}10), (\bar{1}2\bar{1}0), (\bar{2}110)$ and $(2\bar{1}\bar{1}0)$. Intensity variations in both *T* and *R* show that the example of two 24 nm thick graphite rods exhibit dynamical scattering behavior.

Supplementary Notes

Supplementary Note 1. Initial and Reconstructed Probe Functions.

An initial guess for the probe function $P_0(\mathbf{r})$ for the first slice defined at 101 nm below the probe focal point is required for the multislice ptychographic reconstruction using the ePIE algorithm[1]. To obtain this, the experimental amplitude was calculated from an inverse Fourier transform of a Ronchigram taken in the absence of the sample. The phase of the initial probe function, $P_0(\mathbf{r})$, was calculated using the aberration coefficients estimated from the electron-optical microscope alignment[2] as recorded immediately before the data acquisition. Supplementary Fig. 3 shows the initial probe functions (a and b) and the reconstructed probe functions (c and d) recovered for the first slice at $df = 101$ nm using the multislice ptychographic algorithm[3].

Supplementary Note 2. Ewald sphere construction for convergence beam illumination.

The resolution of a ptychographic reconstruction using convergent illumination (Supplementary Fig. 7a can be understood using an Ewald sphere construction. Supplementary Fig. 7b shows the relationship between Ewald spheres for rays incident along the optical axis (—) and at an angle of α (—). The theoretical resolution in this ray arrangement (—) are given by[4];

$$r_x = r_y = \frac{1}{k_{x,\max}} = \frac{\lambda}{\sin(\Theta_{\max})}, r_z = \frac{1}{k_{z,\max}} = \frac{\lambda}{2\sin^2(\Theta_{\max}/2)} \quad (1)$$

where r_x, r_y, and r_z represent spatial resolutions along the x, y, and z directions, respectively, $\Theta_{\max}$ is the maximum scattering angle used in the reconstruction, $k_{x,\max}$ and $k_{y,\max}$ are the maximum lateral distance on the Ewald surface and λ is the electron wavelength.

In our ptychographic experiment as shown in Supplementary Fig. 7a, a convergent incident beam was used and only the central bright-field disk was used for the reconstruction due to the low signal outside the BF disk. For the tilted incident ray (—) at an angle, α relative to the optical axis, the maximum scattering angle within the BF disk that can be used in the reconstruction is twice α as illustrated in Supplementary Fig. 7a. The theoretical resolution in this tilted arrangement can be understood by considering the red Ewald sphere as shown in Supplementary Fig. 7b. The cone of scattered wave vectors (red solid arc) admitted by the BF disk remains constant, which implies that the resolution is improved by a factor of two along the lateral direction and that the resolution along the z axis remains unchanged. Therefore, the overall spatial resolution of the ptychographic reconstruction using the BF disks in the array of diffraction patterns formed using a convergent incident beam with a semi-angle, α can be written as;

$$r'_x = r'_y = \frac{1}{k_{x,\max}} = \frac{\lambda}{2\sin(\alpha)}, r'_z = \frac{1}{k_{z,\max}} = \frac{\lambda}{2\sin^2(\alpha/2)} \qquad (2)$$

As shown in Supplementary Fig. 8, the boundary of the contrast transfer function in reciprocal space consists of four spherical caps (⌒), expressed by;

$$k_z = \begin{cases} \pm\left(\frac{\cos\alpha - \sqrt{1-(\lambda k_x - \sin\alpha)^2}}{\lambda}\right), & k_x \geq 0 \\ \pm\left(\frac{\cos\alpha - \sqrt{1-(\lambda k_x + \sin\alpha)^2}}{\lambda}\right), & k_x < 0 \end{cases} \qquad (3).$$

and hence, for a given spatial frequency k_x the depth resolution is;

$$r'_{z,k_x} = \frac{1}{k_{z,k_x}} = \frac{\lambda}{\left|\cos\alpha - \sqrt{1-(\lambda|k_x| - \sin\alpha)^2}\right|} \qquad (4).$$

Supplementary Note 3. Comparison between Wigner distribution deconvolution (WDD) and inverse-multislice (invMS) ptychography

To demonstrate that the benefit of the invMS compared to the WDD used by Yang *et al.*[5] is explicit handling of multiple scattering that occurs in thick samples, we have carried out a comparison of the optical sectioning results reconstructed using both approaches from simulated diffraction patterns calculated under conditions appropriate to the present experiment using code due to Kirkland[6]. For the sake of computational cost, we have modeled a small area (3 nm × 3 nm) from the overlapping region as shown in Supplementary Fig. 9b. To model the simulated area in the overlapping region, two <0001> orientated single crystalline carbon nano-rods (CNRs) with a graphitic structure and typical AB atomic stacking[7] were constructed. The rods each had a thickness of 24 nm and a spatial separation of 8 nm along the depth direction as shown in Supplementary Fig. 9. The top and side views of the model configurations are shown in Supplementary Figs. 9 b-d, respectively. The red square in Supplementary Fig. 9b indicates the scanning area. The incident electron energy was 60 keV and the probe-forming convergence semi-angle was 22 mrad. Two sets of 22500 simulated diffraction patterns were calculated with both a focused probe for the WDD (Supplementary Fig. 9c) and a probe with a defocus of 28nm for the invMS (Supplementary Fig. 9d), respectively. Each diffraction pattern used a 1024 × 1024 pixel with a sampling of 0.95 mrad per pixel. For comparison, conventional images including BF and ADF were synthesized from the data with collection angles of 0 to 22 mrad and 50 to 180 mrad, respectively as shown in Supplementary Fig. 10 a and b, respectively.

The phase at different depths was subsequently recovered using both the WDD[5] (for a detailed description of this method, see [4]) and the invMS used in this work. Supplementary Figure 11 show 6 slices of phase and amplitude data reconstructed at the top, middle and bottom planes of the two CNRs, using these two methods. The distances of the slices are 0, 12, 24, 32, 44 and 56 nm from the top surface of the upper CNR as shown in Supplementary Fig. 9. Generally, the invMS approach is able to provide optical sectioning images from which the two CNRs can clearly be distinguished at their correct heights. In contrast, the WDD shows optical sectioning images with contrast variations that make it difficult to distinguish the

CNRs when compared to the invMS results. Supplementary Figure 12 shows the enlarged phase reconstructed using the WDD and the invMS at the middle planes of the two CNRs, respectively. It can be seen that the optical sectioning results from the WDD method show a contrast reversal in the phase (Supplementary Figs. 12 a and b) for two CNRs, which suggests this method has been affected by dynamical effects. However, this is not the case for the equivalent phase data reconstructed using the invMS. In addition, by comparing the phases of the lower CNRs, the invMS approach provides more detailed depth-resolved sectioning than the WDD. Therefore, although for 2D imaging the WDD is able to produce results at atomic resolution, its 3D optical sectioning capability is limited by multiple scattering, consistent with the theory describing the WDD, which makes use of the multiplicative approximation.

Supplementary References.

1. Maiden, A. M. & Rodenburg, J. M. An improved ptychographical phase retrieval algorithm for diffractive imaging. *Ultramicroscopy* **109**, 1256-1262 (2009).

2. Sawada, H. *et al.* Measurement method of aberration from Ronchigram by autocorrelation function. *Ultramicroscopy* **108**, 1467-1475 (2008).

3. Maiden, A. M., Humphry, M. J. & Rodenburg, J. M. Ptychographic transmission microscopy in three dimensions using a multi-slice approach. *Journal of the Optical Society of America A-Optics Image Science and Vision* **29**, 1606-1614 (2012).

4. Suzuki, A. *et al.* High-resolution multislice x-ray ptychography of extended thick objects. *Physical Review Letters* **112**, 053903 (2014).

5. Yang, H. *et al.* Simultaneous atomic-resolution electron ptychography and Z-contrast imaging of light and heavy elements in complex nanostructures. *Nature Communications* **7**, 12532 (2016).

6. Kirkland, E. J. *Advanced Computing in Electron Microscopy*. (Springer Science & Business Media, 2010).

7. Jeong, H. K. *et al.* Evidence of graphitic AB stacking order of graphite oxides. *Journal of the American Chemical Society* **130**, 1362-1366 (2008).
